# Sequence mining and transcript profiling to explore cyst nematode parasitism

**DOI:** 10.1186/1471-2164-10-58

**Published:** 2009-01-30

**Authors:** Axel A Elling, Makedonka Mitreva, Xiaowu Gai, John Martin, Justin Recknor, Eric L Davis, Richard S Hussey, Dan Nettleton, James P McCarter, Thomas J Baum

**Affiliations:** 1 Interdepartmental Genetics Program, Iowa State University, Ames, IA 50011, USA; 2 Department of Plant Pathology, Iowa State University, Ames, IA 50011, USA; 3 Department of Molecular, Cellular and Developmental Biology, Yale University, New Haven, CT 06520, USA; 4 Genome Sequencing Center, Department of Genetics, Washington University School of Medicine, St. Louis, MO 63108, USA; 5 Center for Biomedical Informatics, The Children's Hospital of Philadelphia, Philadelphia, PA 19104, USA; 6 Department of Statistics, Iowa State University, Ames, IA 50011, USA; 7 Eli Lilly and Company, Lilly Research Laboratories, Greenfield, IN 46140, USA; 8 Department of Plant Pathology, North Carolina State University, Raleigh, NC 27695, USA; 9 Department of Plant Pathology, University of Georgia, Athens, GA 30602, USA; 10 Divergence Inc., St. Louis, MO 63141, USA

## Abstract

**Background:**

Cyst nematodes are devastating plant parasites that become sedentary within plant roots and induce the transformation of normal plant cells into elaborate feeding cells with the help of secreted effectors, the parasitism proteins. These proteins are the translation products of parasitism genes and are secreted molecular tools that allow cyst nematodes to infect plants.

**Results:**

We present here the expression patterns of all previously described parasitism genes of the soybean cyst nematode, *Heterodera glycines*, in all major life stages except the adult male. These insights were gained by analyzing our gene expression dataset from experiments using the Affymetrix Soybean Genome Array GeneChip, which contains probeset sequences for 6,860 genes derived from preparasitic and parasitic *H. glycines *life stages. Targeting the identification of additional *H. glycines *parasitism-associated genes, we isolated 633 genes encoding secretory proteins using algorithms to predict secretory signal peptides. Furthermore, because some of the known *H. glycines *parasitism proteins have strongest similarity to proteins of plants and microbes, we searched for predicted protein sequences that showed their highest similarities to plant or microbial proteins and identified 156 *H. glycines *genes, some of which also contained a signal peptide. Analyses of the expression profiles of these genes allowed the formulation of hypotheses about potential roles in parasitism. This is the first study combining sequence analyses of a substantial EST dataset with microarray expression data of all major life stages (except adult males) for the identification and characterization of putative parasitism-associated proteins in any parasitic nematode.

**Conclusion:**

We have established an expression atlas for all known *H. glycines *parasitism genes. Furthermore, in an effort to identify additional *H. glycines *genes with putative functions in parasitism, we have reduced the currently known 6,860 *H. glycines *genes to a pool of 788 most promising candidate genes (including known parasitism genes) and documented their expression profiles. Using our approach to pre-select genes likely involved in parasitism now allows detailed functional analyses in a manner not feasible for larger numbers of genes. The generation of the candidate pool described here is an important enabling advance because it will significantly facilitate the unraveling of fascinating plant-animal interactions and deliver knowledge that can be transferred to other pathogen-host systems. Ultimately, the exploration of true parasitism genes verified from the gene pool delineated here will identify weaknesses in the nematode life cycle that can be exploited by novel anti-nematode efforts.

## Background

*Heterodera glycines*, the soybean cyst nematode, is a devastating pathogen of soybean production. Upon hatching as second-stage juveniles (J2), these nematodes migrate through the soil as infective J2, invade roots of soybean plants to become parasitic J2, and move intracellulary through the root tissue until they reach the vicinity of the vascular system, where they become sedentary and induce the formation of a feeding site, the syncytium [1-3]. *H. glycines *completely depends on syncytia for nutrition. Following the development through two more juvenile stages (J3, J4), the nematodes reach adulthood. While adult females remain sedentary, adult males regain motility and leave the root to fertilize females, whose posterior bodies have broken out of the root into the rhizosphere during the course of growth and development. Ultimately, the females die and their body walls harden to protect the eggs, which are mostly retained *in utero*, until the environment is favorable again for a new generation of nematodes to hatch [1,4].

Secreted proteins are key molecular interfaces between parasite and host and enable *H. glycines *to infect soybean plants, which results in an estimated annual damage of $800 million to soybean production in the USA alone [5]. More specifically, secretory proteins that are produced in three large esophageal gland cells (one dorsal and two subventral) and that are injected into host plant cells through the nematode's hollow mouth spear, the stylet, are thought to allow *H. glycines *to migrate through plant tissue by softening and degrading cell walls and to induce and maintain a feeding site, the syncytium, which consists of modified fused host plant root cells. Many genes are involved in adapting cyst nematodes to a parasitic life style. However, only genes whose products are expressed in the nematode secretory glands and are injected into the host cells through the stylet (i.e., parasitism proteins) are called parasitism genes, which in turn have been termed parasitome in their entirety [6-8]. Previous studies in cyst nematodes (*Heterodera *spp. and *Globodera *spp.) identified genes encoding cell wall-degrading and -softening enzymes like beta-1,4-endoglucanase [9,10], pectate lyase [11,12], a putative arabinogalactan endo-1,4-beta-galactosidase [13] and an expansin [14] as members of the parasitome. Apart from genes related to cell wall modifications, other genes whose products likely alter the normal host cell physiology to establish and maintain a syncytium belong to the parasitome, e.g., chorismate mutases [15,16], ubiquitin extension proteins [16,17], as well as S phase kinetochore-associated protein 1 (SKP1) and RING-like proteins [16]. While a few more parasitism proteins have similarity to known proteins, like the venom allergen-like proteins [18] or a chitinase [19], for most *H. glycines *parasitism proteins no clear function can be ascribed [16], although it has been shown that some are imported into plant cell nuclei [20]. To date, more than sixty parasitism genes have been identified in *H. glycines*. Most likely, however, the *H. glycines *parasitome is even larger. Furthermore, there may be proteins produced in organs/tissues other than the esophageal glands or proteins released by means other than the signal peptide-dependent secretory pathway that play critical roles in mediating cyst nematode parasitism. While secretory parasitism proteins that are released into plant host tissue are key factors to understand host-parasite interactions, many secretory proteins exist that do not leave the body of the nematode. These secretory proteins are involved in a vast array of non-parasitic signaling events within the nematode and can be found for example in the extracellular matrix, intestinal lumen, cuticle or neuronal synapse.

Previous studies leading to the identification of parasitism genes in cyst nematodes were based on cloning approaches of single genes [10,11,14,15,17] or exploited smaller scale cDNA libraries constructed from microaspirated gland cell contents [16,21,22]. However, larger scale genomic approaches offer an additional avenue to identify more genes with putative functions in parasitism. While studies to characterize the overall gene expression of parasitic nematodes based on expressed sequence tags (ESTs) have become relatively common in recent years [e.g., [23-32]], very few reports dealt specifically with ESTs of genes that code for secretory proteins [13,33,34], and none combined expression analyses of all genes with detailed sequence mining approaches.

We previously reported the expression profiling of all 7,530 *H. glycines *probesets on the Affymetrix Soybean Genome Array GeneChip representing up to 6,860 unique cyst nematode genes throughout the major life stages from embryonated eggs to the adult female stage [35]. The same Affymetrix GeneChip was used recently to study the expression patterns of a small subset of parasitism genes in a few juvenile life stages of *H. glycines *as well as of soybean genes during the infection process [36] and to survey the development of feeding cells in soybean plants [37]. We generated the necessary cDNA libraries and ESTs that allowed the design of these 7,530 *H. glycines *probesets from stage-specific soybean cyst nematode libraries including not only eggs and infective J2, but also the hard-to-isolate parasitic stages (J3, J4, adult females) that only form inside the soybean root. This latter aspect is particularly important for the research reported here because sedentary endoparasitic nematodes like the cyst nematodes do not produce and/or secrete the vast number of parasitism proteins until they have invaded their host plants. Consequently, previous approaches that are based on preparasitic juvenile worms and that do not include the parasitic stages are liable to miss the truly interesting proteins that the nematode only releases when in contact with its host cell deep inside the plant root. In this current paper, we have analyzed our previously deposited microarray data set [35] with a distinct focus on parasitism and host-parasite interactions. The primary goal of the research presented here was to use critical high throughput criteria discernable from gene sequence and expression characteristics to identify additional proteins that can reasonably be expected to function during parasitism. This pre-selection is an enabling discovery for further, more in-depth functional work to unravel cyst nematode parasitism. To this end, a rigorous examination of all 7,530 *H. glycines *probesets represented on the Affymetrix Soybean Genome Array GeneChip allowed us to identify a pool of 633 *H. glycines *genes that encode putative secretory proteins and of 156 *H. glycines *genes that are conserved in microbes or plants but that have significantly less similarity to sequences from the non-parasitic nematodes *Caenorhabditis elegans *and *Caenorhabditis briggsae*. We provide here for the first time an analysis of the expression profiles of these genes, as well as of all previously described parasitism genes, in all major life stages of *H. glycines *excluding adult males. Together, the gene pool identified here represents a promising starting point to search for previously uncharacterized genes with functions in parasitism and for *H. glycines *genes that potentially were acquired by horizontal gene transfer, a mechanism by which parasitic nematodes are believed to have obtained a subset of parasitism genes [10,38]. In summary, this project represents a functional genomic analysis of available cyst nematode data targeting phytonematode parasitism.

## Results

### Known *H. glycines *parasitism genes show two different developmental expression patterns

**Figure 1:**
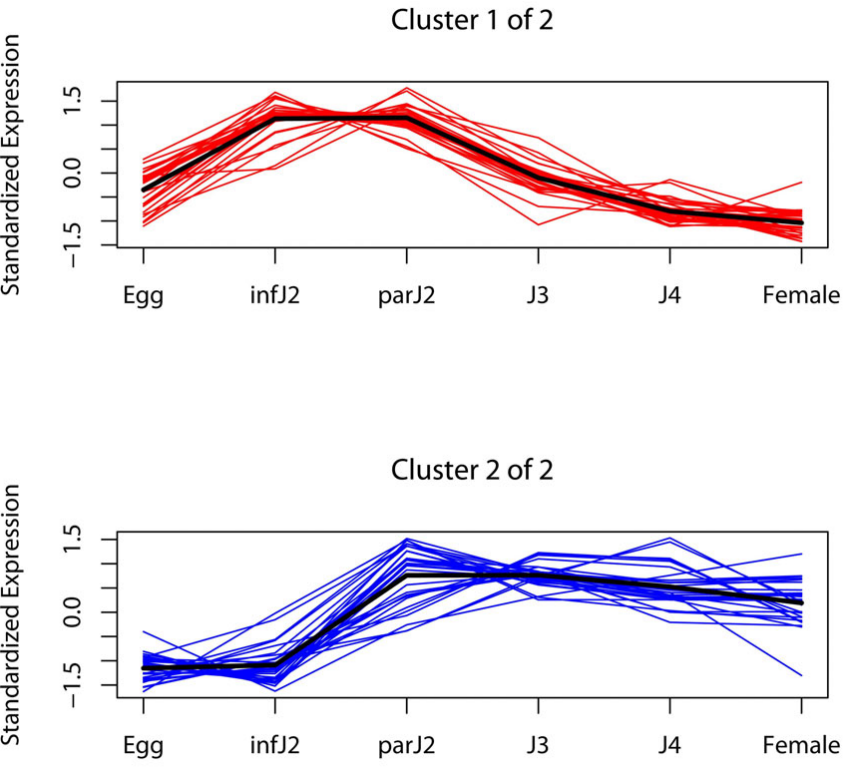
**Temporal expression patterns of *H. glycines *parasitism gene probesets**. All 62 *H. glycines *parasitism gene probesets were differentially expressed (FDR 5%) over the entire life cycle and were grouped into two clusters. The average expression pattern of each cluster is represented by a bold line.

During the generation of the Affymetrix Soybean Genome Array GeneChip the previously identified 66 *H. glycines *parasitism genes [10,12,16,21,22] were grouped by the manufacturer into 46 contigs, which are represented by 62 probesets on the GeneChip (some contigs have multiple probesets) (Additional file 1). Using our previously described Affymetrix microarray data set, which encompasses all life stages of *H. glycines *except adult males [35], we determined the developmental expression patterns of all 62 probesets representing these 46 contigs. All of the parasitism gene probesets displayed significant expression changes during the *H. glycines *life cycle at a false discovery rate (FDR) of 5%. We performed statistical analyses as described in Methods for all parasitism gene probesets, which identified two clusters based on gene expression patterns (Figure 1). *H. glycines *parasitism genes either reached a maximum mRNA abundance in infective J2, remained steady until parasitic J2 and dropped off steeply thereafter (Cluster 1) or they reached a maximum in their mRNA expression levels in parasitic J2 and then fell steadily after the J3 stage (Cluster 2). The gland-specific expression patterns of these genes have been determined in previous studies [16,21,22]. Apart from very few exceptions, parasitism genes expressed in the subventral glands of the nematode fell into Cluster 1, whereas parasitism genes from the dorsal gland could be grouped into Cluster 1 or Cluster 2 (Additional file 1). The majority of *H. glycines *parasitism genes, particularly those expressed in the dorsal esophageal gland, were novel and did not share similarities with any known sequences. Therefore, the expression profiles obtained here are important tools to infer putative protein functions judging from the time points at which these genes are upregulated. Genes that are important during host invasion and early sedentary phases, which includes the early events of syncytium induction (i.e., infective J2/parasitic J2), should be represented in Cluster 1, because this cluster showed an expression maximum in these two stages (Figure 1). Genes that are relevant during later stages of parasitism like the later stages of syncytial development, syncytium maintenance and feeding, are expected in Cluster 2, whose members were expressed at a relatively high level also in late stages (J3, J4, adult females). However, it was remarkable that such diverse proteins as secreted cellulases and pectate lyases with an obvious role in plant cell wall degradation (i.e., during early phases of parasitism) had a strikingly similar expression pattern to genes encoding secretory proteins that are believed to influence the host cell's physiology (e.g., chorismate mutases, ubiquitin extension proteins) or for which no function during parasitism can be ascribed yet (e.g., chitinase, venom allergen proteins). The expression profiles of these genes showed a clear downregulation after the parasitic J2 stage, which is the beginning of the sedentary phase of the nematode and of feeding site development. Furthermore, those parasitism genes that were upregulated in later life stages did not match any other known sequences, apart from two exceptions: annexin and a cellulose-binding protein (Additional file 1). Annexins bind to calcium-dependent phospholipid membranes, and while their role during parasitism remains unknown, the cellulose-binding protein binds to a plant pectin methylesterase and functions in cell wall modifications during syncytium development [39].

In addition to analyses of gene expression changes throughout the whole life cycle, in which each life stage was compared to all others, we also performed statistical analyses to identify gene expression changes in consecutive life stages to isolate specific shifts in gene expression. The results of these studies showed that most parasitism genes were differentially expressed when eggs were compared with infective J2 as well as when infective J2 were compared with parasitic J2. All of these differentially expressed genes were upregulated (Table 1). This pattern drastically changed from parasitic J2 to J3, where only a smaller subset of genes was differentially expressed and, interestingly, these were all down-rather than upregulated.

**Table 1:** Summary of parasitome, signal peptide-encoding and plant or microbe-like *H. glycines *genes.

***H. glycines *contigs**	**Contigs**	**Probesets**	Differentially expressed probesets total (up/downregulated)**					
			**overall life cycle**	**egg/infJ2**	**infJ2/parJ2**	**parJ2/J3**	**J3/J4**	**J4/F**
Secretory protein coding*	633	686	646	337 (213/124)	335 (186/149)	224 (55/169)	69 (41/28)	136 (41/95)
***H. glycines* parasitome**	46	62	62	39 (34/5)	34 (30/4)	34 (0/34)	2 (0/2)	3 (0/3)
**Plant-like**	29	30	18	2 (0/2)	4 (4/0)	1 (1/0)	0	2 (0/2)
**Phytomicrobe-like**	41	43	28	16 (9/7)	7 (5/2)	3 (1/2)	5 (5/0)	3 (1/2)
**Soil microbe-like**	33	40	25	11 (4/7)	7 (4/3)	3 (1/2)	0	4 (4/0)
**Other microbe-like**	53	63	39	11 (5/6)	14 (11/3)	5 (2/3)	0	5 (0/5)

*As defined in Methods

**FDR 5%

### *H. glycines *genes for secretory proteins frequently are novel sequences and change expression with the onset of parasitism

To identify new *H. glycines *genes that are potentially involved in host-parasite interactions we identified all genes that encode secretory proteins. Specifically, we searched for signal peptide-coding regions in the consensus sequences of all 7,530 *H. glycines *probesets on the Affymetrix Soybean Genome Array GeneChip as described in Methods. This analysis identified 633 unique *H. glycines *genes that encode proteins with a putative signal peptide but lack a transmembrane helix and have an open reading frame (ORF) of at least thirty amino acids after the predicted signal peptide cleavage site (Additional file 2). However, it can be assumed that *H. glycines *possesses more gene products with signal peptides than we could detect here because a significant portion of ESTs lacks a complete 5'-end so that no signal peptide-coding region could possibly be identified. All known parasitism genes are included in these 7,530 probesets, and our detection protocol for secretory protein coding genes re-identified all but seven of the known sixty-six parasitism genes analyzed here, giving a re-discovery rate of 89%. The probesets of genes that were missing did not meet all of our stringent selection criteria.

Because most parasitism proteins have no database hits to known proteins, we determined whether the signal peptide-encoding genes identified here differ in their likelihood of having database matches compared to other genes. For this purpose, we conducted BLASTX searches of all 6,860 unique *H. glycines *genes underlying the 7,530 probesets of the Affymetrix GeneChip against the non-redundant GenBank database. We found that 52.8% (334/633) of the gene products predicted to be secreted had matches when using a cutoff value of 1e-05 (Additional file 2). Similarly, 58.5% (3,644/6,227) of the gene products not thought to be secreted had matches. Even though this difference of 5.7% is relatively small, we found a significant difference when the relative BLASTX scores were compared. Out of all 6,860 unique genes, 3,978 had matches, and resulted in a median BLASTX score of 310 (min 41, max 5,249). Using only the 334 gene products of secreted candidates, the median BLASTX score was reduced to 129 (min 27, max 3,299), which suggests that as was the case for previously reported parasitism genes, our newly identified cohort of secretory proteins containing potential parasitism proteins evolved more rapidly than non-secreted sequences.

**Figure 2:**
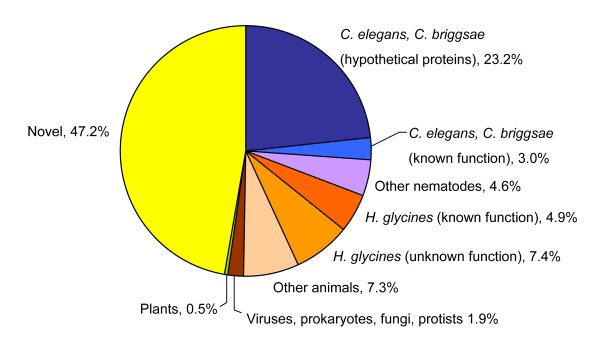
**BLASTX hits of *H. glycines *putative secretory protein encoding genes separated by organism group**.

To analyze with which organisms *H. glycines *signal peptide-bearing proteins share conservation, we sorted the top BLASTX hits meeting a cutoff of 1e-05 by organism (Figure 2). Approximately 26% (166 genes) of the 633 *H. glycines *genes with putative signal peptide-coding regions matched *Caenorhabditis elegans *or *Caenorhabditis briggsae *sequences, followed by 12% that matched other *H. glycines *genes and 7% that aligned best with sequences from animals other than nematodes. However, 147 out of the total 166 *Caenorhabditis *matches represented unknown or hypothetical proteins and, similarly, 47 out of 78 *H. glycines *sequences were novel, i.e., they did not share similarity with other known sequences.

**Figure 3:**
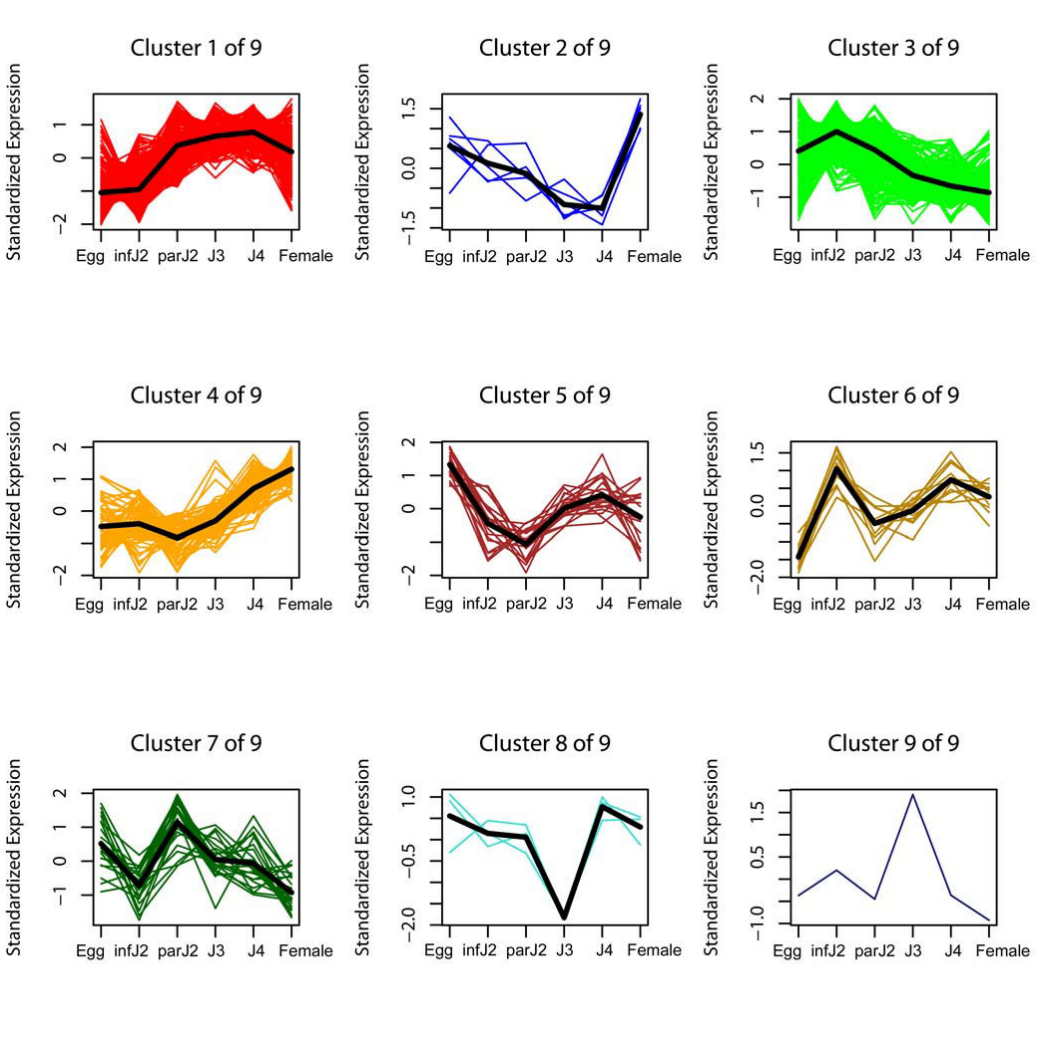
**Temporal expression patterns of probesets for predicted *H. glycines *secretory protein encoding genes**. All 646 *H. glycines *probesets encoding putative secretory proteins with predicted signal peptide, at least thirty amino acids after the signal peptide cleavage site and no predicted transmembrane helix were differentially expressed (FDR 5%) over the entire life cycle and were grouped into nine expression clusters. The average expression pattern of each cluster is represented by a bold line.

Using statistical analyses, all Affymetrix probeset expression profiles obtained for the 633 signal peptide-encoding genes separated into nine expression clusters (Figure 3) as described in Methods. When all experimental data, i.e., expression data throughout all life cycle stages, were taken into consideration, 94% of these probesets were differentially expressed (FDR 5%) during the life cycle. When conducting statistical analyses of expression data for consecutive life stages in pairwise comparisons, we determined that 49% of the Affymetrix probesets representing the 633 secretory protein genes were differentially expressed (FDR 5%) during the transition from eggs to infective J2. Similarly, 48% of these Affymetrix probesets were differentially expressed from infective J2 to parasitic J2 and 33% from parasitic J2 to J3. The fewest significant changes (10%) occurred between J3 and J4, followed by the comparison between J4 and adult females (20%) (Table 1). Taken together, the clusters for overall differentially expressed probesets as well as the stage by stage comparisons showed that the strongest expression changes in genes that code for secretory proteins took place at the transitions into and out of the infective J2 stage, which marks the preparations for host invasion and a parasitic lifestyle.

### Identification and expression profiling of *H. glycines *genes that are conserved in microbes or plants

Previous studies showed that a substantial proportion of the known cyst nematode parasitism proteins share a high degree of similarity with bacterial [10,11,13] and fungal [11-13] proteins and not to the non-pathogenic *Caenorhabditis *spp. These observations led to the suggestion that certain nematode genes that are important for a parasitic relationship with the host might have been acquired by horizontal gene transfer from microbes [7,10,38]. Further studies demonstrated that other cyst nematode secretory proteins with potential involvement in parasitism have a striking similarity to plant proteins [15,16,40,41], which could hint at mimicry of plant regulatory proteins by cyst nematodes to alter the physiology of the host. Therefore, we identified *H. glycines *proteins that showed significant sequence similarity with microbe or plant proteins by manually sorting the results of the BLASTX searches of all 6,860 *H. glycines *genes against the non-redundant GenBank database mentioned above. We isolated all matches to proteins from plants, microbial phytopathogens/phytosymbionts (termed phytomicrobes here), soil-living microbes and other microbes. A counter-selection protocol ensured that highly conserved sequences that are present in diverse organisms (including *Caenorhabditis *spp.) were removed in order to discard sequences that are unlikely to be involved in parasitic relationships (see Methods). These analyses revealed that, using our criteria, 29 *H. glycines *protein sequences were conserved in plants, 41 in microbial phytomicrobes, 33 in soil-living microbes, and 53 in other microbes, resulting in a total of 156 such proteins (Additional files 3, 4, 5, 6). For example, we identified a plant-like *H. glycines *gene whose translated product had similarity to beta-amylase from Arabidopsis (HgAffx.12554.1), which is interesting because genes encoding beta-amylases have so far only been found in microbe and plant genomes, but not in animals. Furthermore, we found *H. glycines *genes whose products were similar to a potato protein induced in the feeding site (giant-cells) of the root-knot nematode (*Meloidogyne*) (HgAffx.13422.1), or were involved in RNA interference (RNAi) like a Zwille/Pinhead-like protein from rice (HgAffx.13330.1). Phytomicrobe-like *H. glycines *sequences matched sugar metabolizing enzymes from *Agrobacterium tumefaciens*, e.g., mannitol-2-dehydrogenase (HgAffx.18502.1) and sucrose hydrolase (HgAffx.9663.1, HgAffx.12954.1), as well as enzymes like glutamine synthetase (HgAffx.18955.1) and phosphoribosyltransferase (HgAffx.23512.1) or aldose-1-epimerase (HgAffx.18360.1) from *Mesorhizobium *to name just a few.

It is particularly interesting to discern if these *H. glycines *genes potentially are secreted from the nematode. Therefore, we examined how many of these *H. glycines *genes were part of the secretome identified in this study. Of the 29 plant-conserved *H. glycines *genes, three encoded a putative signal peptide. Similarly, four of those conserved in phytomicrobes, four of those conserved in soilmicrobes and four in other microbes (Additional file 7) contained signal peptide sequences. However, these predicted proteins did not necessarily meet all our other, more stringent criteria. E.g., some proteins did have a putative transmembrane helix or did have less than 30 amino acids after the predicted signal peptide cleavage site (see Methods). Also in these analyses, one needs to remember that a significant portion of the ESTs lacks a complete 5'-end so that no signal peptide-coding region could possibly be detected. Additional file 7 shows the overlap between *H. glycines *contigs identified as parasitism genes, secretory protein-encoding or plant/microbe-like. Interestingly, some of those *H. glycines *genes that did fulfill all our criteria for secreted plant-like proteins matched for example plant genes encoding histone deacetylase 2 from Arabidopsis (HgAffx.19783.1) or the *Meloidogyne*-induced giant-cell protein-like protein from potato (HgAffx.13422.1). *H. glycines *proteins with similarity to microbial gene products that passed our signal peptide selection process matched among others a flavin adenine dinucleotide (FAD)-linked oxidase from *Arthrobacter *(HgAffx.18477.1), histidine triad nucleotide-binding family protein 1 (HIT1) from *Chaetomium globosum *(HgAffx.11878.1) or a hypothetical protein from *Gibberella zeae *(HgAffx.17226.1). To our knowledge, none of these genes encode proteins with signal peptides in the respective plant or microbe species to which the *H. glycines *sequences matched.

We further grouped all identified plant- and microbe-like *H. glycines *genes represented by their respective probesets into distinct expression clusters (Additional files 8, 9, 10, 11). In order to identify specific shifts in gene expression, we performed pairwise comparisons of consecutive life stages of *H. glycines *for genes that were conserved in plants, phytopathogens/phytosymbionts, soilmicrobes and other microbes regardless of whether we could find a signal peptide-encoding sequence or not. The results of these analyses are summarized in Table 1. It is evident that the vast majority of these probesets was differentially expressed when eggs were compared with infective J2 and infective J2 with parasitic J2 and that up- and downregulated probesets were represented in about equal proportions in these two stage-wise comparisons. This means that these plant and microbe-like *H. glycines *genes are strongly regulated at the onset of parasitism.

### *H. glycines *encodes signal peptide-bearing gene products with similarity to plant histone deacetylase

Histone modifications are an important regulatory element in gene expression [42]. We found here a *H. glycines *histone deacetylase-2 (HDA2) probeset (HgAffx.19783.1.S1_at) with an Arabidopsis HDA2 (AAM34784.1) as best BLASTX match for its consensus sequence, rather than a homologous gene in nematode species, including the fully sequenced *C. elegans *or *C. briggsae *genomes. Interestingly, this *H. glycines *HDA2 has a putative signal peptide and lacks a transmembrane helix, which makes it a putatively secreted protein. To our knowledge, there are no prior reports of signal-peptide-containing histone deacetylases. Cyst nematodes like *H. glycines *are known to possess plant-like proteins with signal peptides that are normally not secreted in plants or other organisms, e.g., SKP1 or chorismate mutases [7]. Hence, a secreted Arabidopsis-like histone deacetylase would be an exciting new example of how cyst nematodes could modify gene expression of plants by altering the epigenome of their host cells.

**Figure 4:**
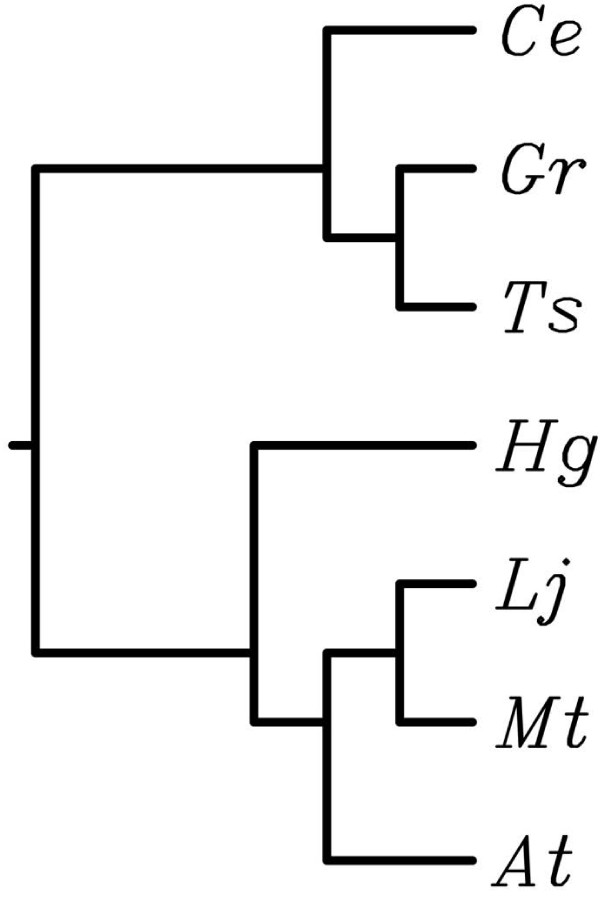
**CLUSTAL W phylogenetic tree for HDA2 homolog sequences**. Including *H. glycines *(Hg) Affymetrix consensus sequence for probeset HgAffx.19783.1.S1_AT; *C. elegans *(Ce) Wormbase entry CE01472; *Trichinella spiralis *(Ts) ES567375; *Globodera rostochiensis *(Gr) AW506399; *Arabidopsis thaliana *(At) AAM34784.1; *Medicago truncatula *(Mt) EV261025; *Lotus japonicus *(Lj) BW625394.

To analyze this *H. glycines *gene product further, we constructed a multiple alignment and a phylogenetic tree for the translated Affymetrix consensus sequence of this probeset (HgAffx.19783.1.S1_at) and HDA2 homologs of selected plant and nematode species using CLUSTAL W [43]. It can be clearly seen that the *H. glycines *HDA2 is not only more similar to Arabidopsis HDA2, but to other plant HDA2s as well, and that the respective homologs of other nematode species are very different from plant HDA2s (Figure 4, Additional file 12).

### *H. glycines *probes cross-hybridize with *Phytophthora sojae *or *Glycine max *probesets

**Figure 5:**
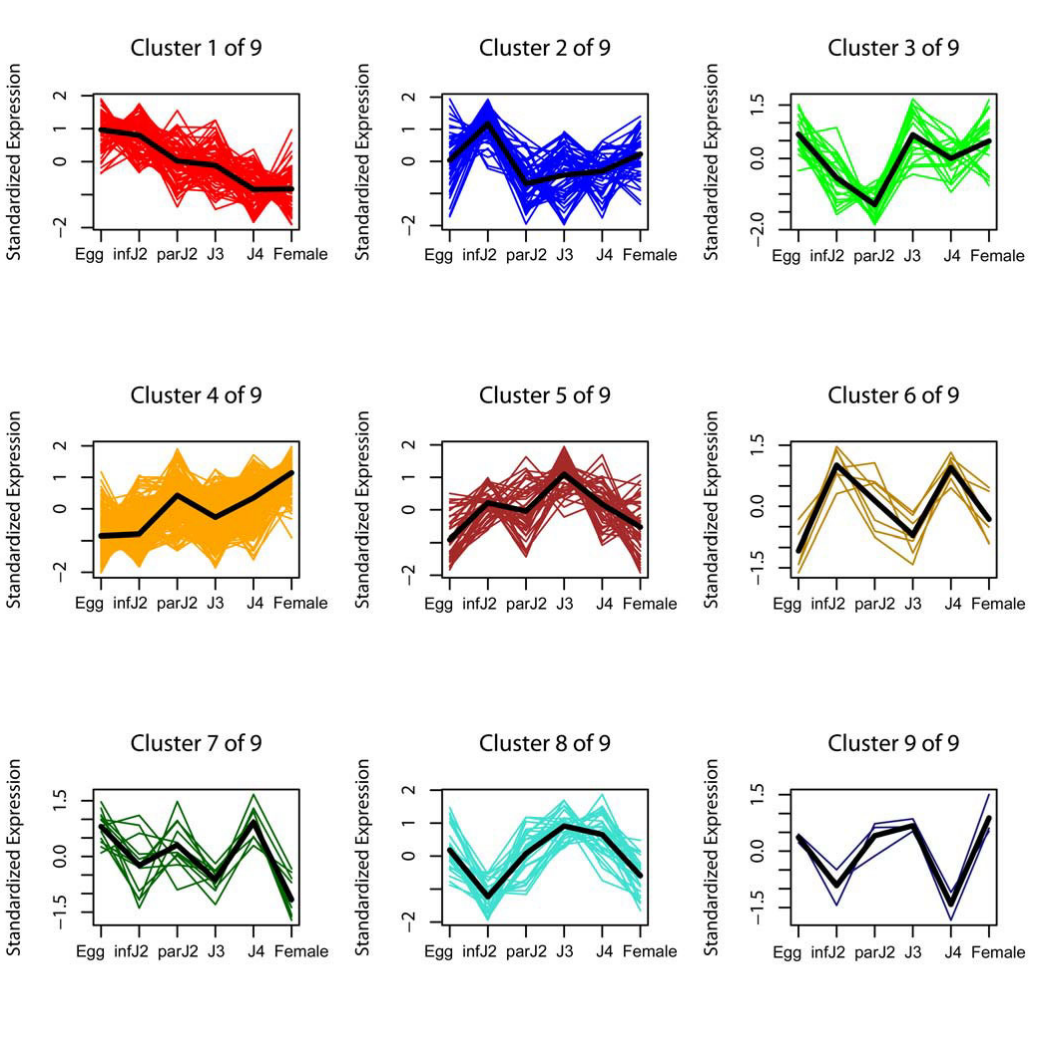
**Temporal expression patterns of cross-hybridizing soybean probesets**. We identified 576 soybean probesets that cross-hybridized to *H. glycines *probes. These probesets were grouped into nine expression clusters. The average expression pattern of each cluster is represented by a bold line.

**Figure 6:**
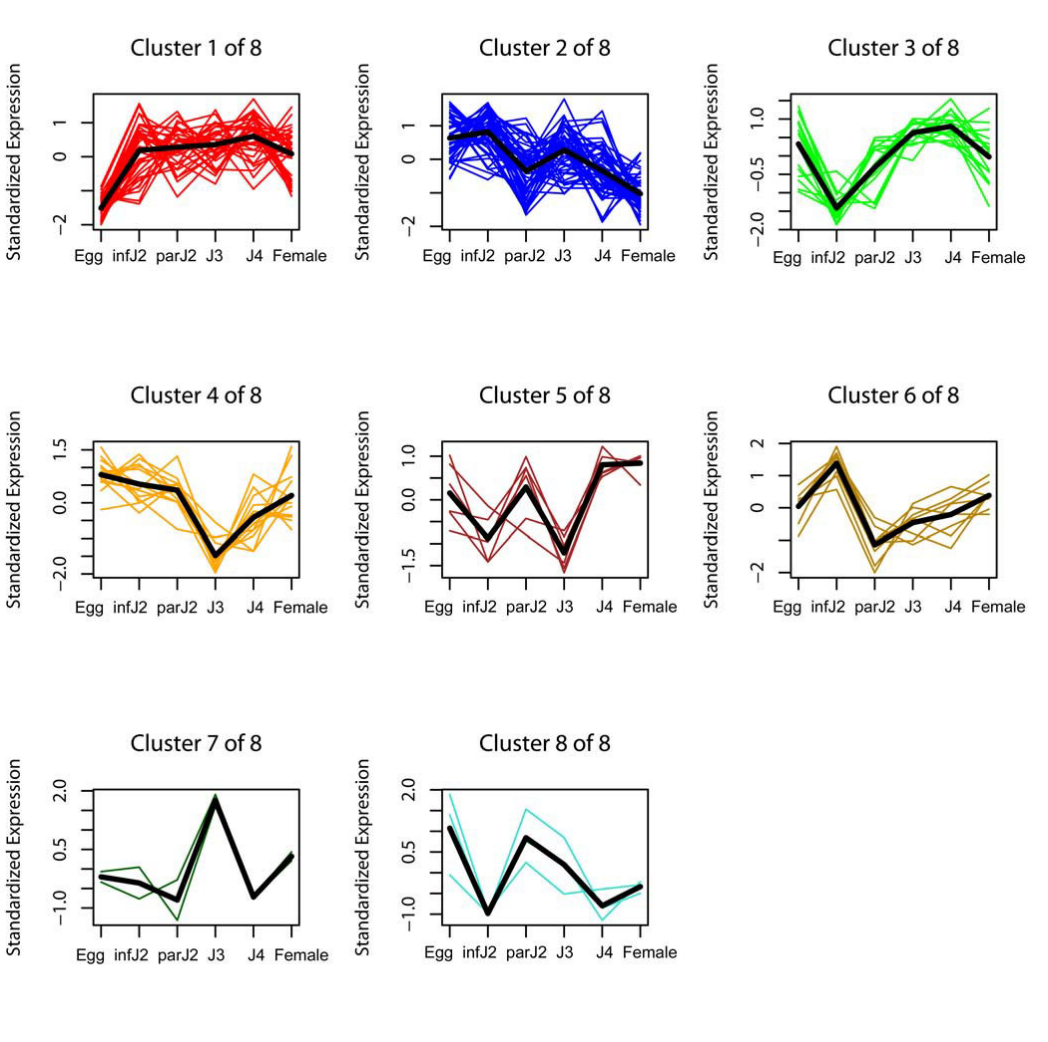
**Temporal expression patterns of cross-hybridizing *P. sojae *probesets**. We identified 134 *P. sojae *probesets that cross-hybridized to *H. glycines *probes. These probesets were grouped into eight expression clusters. The average expression pattern of each cluster is represented by a bold line.

In addition to the 7,530 probesets corresponding to *H. glycines *mRNAs, the Affymetrix Soybean Genome Array GeneChip contains 37,500 probesets for soybean (*Glycine max*) mRNAs and 15,800 probesets for mRNAs of *Phytophthora sojae*, an oomycete pathogen of soybean plants. We found 576 *G. max *and 134 *P. sojae *probesets that hybridized strongly and repeatedly to *H. glycines *probes in three independent experiments (Additional files 13, 14) and that showed differential expression (Figures 5, 6). A variety of BLAST searches of cross-hybridizing probesets (BLASTX, BLASTN against non-redundant GenBank; BLASTN against dbEST_other database) detected only a negligible number of *Caenorhabditis *hits and did not lead to matches for cyst nematode sequences, but primarily soybean and oomycete or fungus sequences as best hits (data not shown). These results indicate that the cross-hybridizing probesets in all likelihood originated from soybean plants and *P. sojae *and were not *H. glycines *contaminations in cDNA libraries of these two organisms because we did not find a single hit with a *bona fide H. glycines *database entry, which otherwise would have to have occurred. The possibility of falsely annotated *H. glycines *ESTs can be ruled out based on the same results. To determine whether in fact there are highly conserved cyst nematode sequences that could cross-hybridize with gene sequences of soybean or *P. sojae*, we conducted BLASTN searches of the cross-hybridizing soybean and *P. sojae *probesets against an in-house database of all cyst nematode (*Heterodera *spp. and *Globodera *spp.) sequences. Of 576 soybean probesets, 119 (20.7%) indeed matched known cyst nematode genes and of 134 *P. sojae *probesets 12 (9.0%) in fact had matches with known *Heterodera *spp. or *Globodera *spp. genes (Additional files 13, 14). In other words, in all likelihood, sequence conservation was responsible for the observed cross-hybridization results.

To complement the BLAST searches of the cross-hybridizing soybean and *P. sojae *probesets, we used InterProScan [44] to identify known protein domains. We found 336 soybean probesets that aligned to 207 unique InterPro domains and 83 *P. sojae *probesets that aligned to 70 unique InterPro domains, respectively. A summary of the 25 most abundant InterPro domains for both organisms can be viewed in Table 2.

**Table 2:** The 25 most abundant InterPro domains for *G. max *and *P. sojae *probesets that cross-hybridized with *H. glycines *probes.

*G. max*			*P. sojae*		
InterPro domain		Number of probesets	InterPro domain		Number of probesets
Ubiquitin	IPR000626	16	Sugar transporter superfamily	IPR005829	3
Extensin-like protein	IPR003883	9	EGF-like region	IPR013032	3
Glutamine amidotransferase	IPR000583	9	Peptidase S8 and S53	IPR000209	2
ATP-sulfurylase	IPR002650	8	HMG-I and HMG-Y, DNA-binding	IPR000637	2
Histone-fold	IPR009072	6	TonB box, N-terminal	IPR010916	2
Zinc finger, C2H2-type	IPR007087	6	Penicillin-binding protein	IPR012338	2
Major intrinsic protein	IPR000425	5	Rhodopsin-like GPCR superfamily	IPR000276	2
EGF-like region	IPR013032	5	DEAD/DEAH box helicase	IPR001410	2
DNA-binding WRKY	IPR003657	4	Prefoldin	IPR009053	2
Cytochrome c oxidase	IPR000883	4	Helix-turn-helix, Fis-type	IPR002197	2
Cupredoxin	IPR008972	4	Zinc finger, C2H2-type	IPR007087	2
Bet v I allergen	IPR000916	3	Ctr copper transporter	IPR007274	1
Chlorophyll A-B binding protein	IPR001344	3	Glycoside hydrolase	IPR001764	1
Orn/DAP/Arg decarboxylase 2	IPR000183	3	Zinc finger, RING-type	IPR001841	1
No apical meristem (NAM) protein	IPR003441	3	Protein prenyltransferase	IPR002088	1
IQ calmodulin-binding region	IPR000048	3	Whey acidic protein	IPR008197	1
Zinc finger, RING-type	IPR001841	3	Histone-fold	IPR009072	1
BURP	IPR004873	3	Ribosomal protein L10	IPR001790	1
Ribosomal protein L7Ae/L30e/S12e/Gadd45	IPR004038	3	Major facilitator superfamily MFS_1	IPR011701	1
UspA	IPR006016	3	Phosphotransferase KptA/Tpt1	IPR002745	1
Ribosomal protein L32e	IPR001515	3	Regulator of chromosome condensation	IPR000408	1
Dehydrin	IPR000167	3	Inositol 1, 3, 4-trisphosphate 56-kinase	IPR008656	1
Thioredoxin-like fold	IPR012336	3	Serine carboxypeptidase	IPR001563	1
Thiamine pyrophosphate enzyme	IPR011766	3	Na+ solute symporter	IPR001734	1
DNA photolyase	IPR005101	2	Fructose-bisphosphate aldolase	IPR000741	1

*As defined in Methods

**FDR 5%

Taken together, the soybean and *P. sojae *probesets that surprisingly cross-hybridized with *H. glycines *probes potentially provide a lead to an additional set of nematode genes from which novel parasitism-associated genes can be isolated and confirmed. Ultimately, this aspect can be advanced further only with a future release of a complete *H. glycines *genome sequence, because the recent deposition of a large number of genome fragments did not allow an exhaustive and conclusive analysis (data not shown).

## Discussion

Here we have presented the results of an extensive *in silico *study aiming at the identification of a group of *H. glycines *genes that is enriched in genes with potential functions during host-parasite interactions. This is the first study combining microarray data of all major life stages (except adult males) and exhaustive sequence analyses for the identification of parasitism-associated proteins in any parasitic nematode. Our findings now enable more detailed studies to identify true parasitism genes among the individual candidate genes identified here, which would not have been feasible for larger data sets.

It has been widely assumed that cyst nematode parasitism genes have well-defined roles in early and late stages of the infection process, and our findings of two distinct expression clusters representing early or late upregulation for all known parasitism genes support this. When comparing the temporal expression patterns of *H. glycines *gene groups identified here, it became evident that certain expression clusters showed profiles that were similar to those of known parasitism genes. Specifically, Cluster 1 of the currently known parasitome (Figure 1) (i.e., early upregulation) was strikingly similar to Cluster 3 of the secretome identified by us here (Figure 3), while Cluster 2 of the parasitome (i.e., late upregulation) was mirrored by Cluster 1 of the secretome, Cluster 1 of plant-like *H. glycines *genes (Additional file 8) and Cluster 5 of soilmicrobe-like *H. glycines *genes (Additional file 10). The *H. glycines *genes represented in these clusters might be particularly promising candidates for future studies aiming at the verification of their roles in parasitism. Alternatively, Clusters 2 and 4 of the secretome might be of interest because the genes in these groups were strongly upregulated in very late parasitic stages (post-J3). These clusters might harbor novel parasitism-associated genes that are very different from the currently known parasitome, which is biased towards early stages of parasitism.

It has been suggested that nematode proteins that enter the secretory pathway evolve more rapidly than those that do not and hence are less likely to match sequences of other organisms [33]. Even though the difference we found in the number of genes with BLASTX hits between all *H. glycines *genes represented on the Affymetrix microarray and those with a predicted signal peptide was relatively low, we identified a significant difference in the relative distribution of BLASTX scores. This means that our results support previous findings and a model in which nematode secretory proteins evolve more rapidly than other gene products. None of the libraries used here was based on splice leader 1 (SL1) sequences, which tend to be skewed towards shorter sequences [24], such that no bias towards complete 5'-ends (and hence signal peptides) was possible in the data analyzed here.

Some of the known parasitism proteins showed similarities to plant or microbe sequences [10-12,14,16], and in a closely related nematode species additional genes with similarity to nitrogen-fixing soil bacteria, collectively called rhizobia, could be identified [38]. It has been suggested that microbial genes might have been acquired by horizontal gene transfer [10,38] and that plant-like genes might have evolved in the nematode for mimicry of plant proteins and interference with plant signaling pathways [7,40,41]. For all of these genes to have any effect in a parasitic relationship between host and parasite, they need to be secreted. Hence, we were interested in analyzing whether *H. glycines *genes represented on the Affymetrix Soybean Genome Array GeneChip with similarity to microbes or plants might encode signal peptide-bearing proteins. If so, these genes would be interesting candidates for further studies as they might be involved in parasitism. Particularly intriguing examples were genes encoding proteins for which secretion would not be expected in plants or microbes, but for which homologous sequences in *H. glycines *possessed a signal peptide-encoding region. For example, we found two *H. glycines *genes (HgAffx.19783.1, HgAffx.19046.1) with similarity to Arabidopsis histone deacetylase 2 (AAM34784.1) and Arabidopsis RNA polymerase II transcription factor (NP_175948.1), none of which are secreted in Arabidopsis, but both contained a signal peptide in *H. glycines *(Additional file 12). Given the fact that these proteins had higher similarity with plant proteins than with homologs in the fully sequenced *C. elegans *or *C. briggsae *genomes, secretion of these proteins into host plants and a role in parasitism seems very likely and warrants further experiments. It has been demonstrated that histone modifications are involved in gene regulation [42] so that a secreted Arabidopsis-like histone deacetylase would be an exciting example of how cyst nematodes could modify gene expression of their host plants and mimic plant proteins to interfere with the physiology of the host. It has been shown that in Arabidopsis, histone acetylation is involved in vernalization [45], is responsive to light [46], and that histone deacetylase 19 (HDA19) can be induced by wounding, pathogen attack and plant hormones [47]. Furthermore, overexpression of HDA19 resulted in increased pathogen resistance [47]. What role a secreted *H. glycines *histone deacetylase might have during the infection process remains elusive at this point.

As stated earlier, the sequences upon which the Affymetrix GeneChip is based are mostly ESTs, which frequently are incomplete at their 5'-ends, which is the location of the signal peptide-encoding sequence. Consequently, we expect that more of the plant and microbe-like *H. glycines *genes encode secretory proteins than we were able to identify. On the other hand, not all of the *H. glycines *proteins that matched plant or microbe sequences and that did have a signal peptide are necessarily secreted into the environment (i.e., the host plant) but are rather involved in processes within the nematode. Further experimental studies are needed to localize the putative secretory proteins identified here in the nematode either by in situ hybridizations or immune localizations. Also, it needs to be noted that certain proteins can be secreted even without a canonical N-terminal signal peptide [48], which adds an additional layer of complexity to the study of cyst nematode parasitism.

Our findings of *H. glycines *genes with similarity to plant or microbe sequences identified here do not imply that all these genes have been acquired by horizontal gene transfer or have evolved to mimic host proteins. The set of genes identified here is rather meant as a first step to identify a pool of candidates from which true parasitism-related genes can be isolated from highly conserved, but not parasitism-related ones, in the future. Interestingly, recent studies have demonstrated that cellulase genes must have been present in an ancient ancestor of bilaterian animals [49,50] and, therefore, may not have been acquired by nematodes through horizontal gene transfer.

A direct demonstration of *H. glycines *genes that are conserved in microbes and plants could be seen in the *P. sojae *and soybean probesets of the Affymetrix Soybean Genome Array GeneChip to which *H. glycines *probes cross-hybridized in our experiments. While many of the genes had highly conserved functions like DNA-binding domains, sodium symporters or zinc fingers, others could possibly be involved in parasitic relationships between *H. glycines *and soybean plants. Interestingly, cross-hybridizing *G. max *probesets matched ten distinct *H. glycines *sequences that were derived from esophageal gland cell cDNA libraries, and *P. sojae *matched two, respectively (Additional files 13, 14). The respective *H. glycines *genes are of unknown function, such that a putative role in parasitism is speculative at this point. It is extremely unlikely that the cross-hybridizing probesets are caused by contaminating soybean or oomycete nucleic acids. For one, even if there were plant material left after our nematode isolations, the amounts would be so minute that repeatedly strong signals at about the same level and same developmental stage of the nematode in the different experiments performed by us are highly unlikely. Even more compellingly, we harvested *H. glycines *eggs in a very pure state and infective J2 stages from hatch chambers, a plant-free environment. Both stages show strong expression signals for many soybean probesets. Since our BLAST searches described in Results ruled out the possibility of falsely annotated nematode sequences in *P. sojae *or soybean cDNA libraries, we believe that the strong expression signals for cross-hybridizing soybean and *P. sojae *probesets must originate from homologous genes in *H. glycines*.

## Conclusion

In summary, we have identified a novel pool of putative parasitism-associated genes, a significant proportion of which, we hypothesize, will turn out to have parasitic functions after functional assays have been performed. We also raise the possibility that *H. glycines *might have acquired many more genes through horizontal gene transfer and might mimic many more plant proteins, all of which could be involved in parasitism, than previously thought. Using powerful genomic tools, this study has reduced the total number of 6,860 currently known *H. glycines *genes to a pool of 788 candidate genes, from which additional true parasitism genes can be identified in future studies. The identification of these candidate genes is a very significant advance for the field, but also of broad interest for pathogen-host research in general because this new pool of genes will help unravel sophisticated plant-animal interactions leading to a successful parasitic relationship and deliver knowledge that can be transferred to other pathogen-host systems. Ultimately, the verification of true parasitism genes from the pool of candidate genes isolated here and their subsequent functional characterization will identify weaknesses in the nematode life cycle that can be targeted in novel anti-nematode efforts.

## Methods

### Nematode cultivation

The microarray data analyzed here is based on our previously published experiments [35]. Briefly, for that study, we planted forty pots of Kenwood 94 soybean seed under greenhouse conditions in three replications and all nematodes used within a given replication were from the same experimental setup and the same batch of eggs. Two weeks after planting, each pot was inoculated with 15,000–20,000 *H. glycines *strain OP-50 [51] infective J2. The inoculum was collected from 4 day old hatch chambers, each containing about two million *H. glycines *OP-50 eggs. From the same batch of eggs used in the hatch chamber, 50,000 eggs were collected and flash frozen in liquid nitrogen, for use as the egg stage hybridization probe. After four days, an aliquot of 50,000 hatched infective J2 was flash frozen in liquid nitrogen, for use as the infective J2 stage probe. The remainder of hatched infective J2 was divided among the forty pots for seedling inoculation. Four days after infection, parasitic J2 were collected from twelve pots. Eight days after infection, another twelve pots were harvested for collection of J3 juveniles and fourteen days after infection, a further ten pots were used to collect J4 juveniles. Finally, twenty-one days after infection, the final six pots were harvested for collection of adult females.

### RNA extraction and GeneChip hybridization

All data analyzed here is based on our previous results [35]. For that study, frozen nematode tissue was disrupted with frozen zirconia beads (BioSpec Products, Bartlesville, OK) in a beadbeater (BioSpec Products, Bartlesville, OK). RNA was isolated using the Versagene kit (Gentra Systems, Minneapolis, MN) as described [35]. RNA quality and concentration of each sample were determined by RNA Nanochip on a 2100 Bioanalyzer (Agilent Technologies Inc, Palo Alto, CA) and by a NanoDrop spectrophotometer (NanoDrop Technologies, Wilmington, DE). Standard procedures for reverse transcription and labeling of the probes and for hybridization and scanning of the GeneChips were followed by the Iowa State University GeneChip Facility.

### Design of microarray experiments and GeneChip data analysis and validation

For our previous study [35], from which the microarray data analyzed here has been drawn, we measured expression using 18 Affymetrix Soybean Genome Array GeneChips (3 replications × 6 life stages) using a randomized complete block design with replications as blocks. In that study, the Affymetrix signal data were transformed with the natural log (ln) and normalized by median centering prior to the analysis. The normalized data for each gene were analyzed separately using a standard linear model with fixed effects for replications and stages. To test for a difference in expression between life stages for each probeset, we performed F tests, which resulted in p-values. A q-value was calculated for each p-value following [52] and was used to maintain approximate control of the false discovery rate (FDR) at 5% by declaring q-values at or below 0.05 significant. For a detailed description of biological sample preparation, RNA extraction and experimental design see our previous study [35] and [53].

Clustering was implemented to group the observed expression patterns of differentially expressed genes. We estimated the mean normalized expression level for each probeset in all six life stages. The resulting six estimated values were standardized to have mean 0 and standard deviation 1 within each probeset. The Euclidian distance between any pair of standardized expression profiles was used as a measure of dissimilarity in all clustering algorithms. This approach considers genes with similar expression patterns to be close in six-dimensional space and is equivalent to using (1-r)^0.5 ^as the measure of dissimilarity, where r is the Pearson correlation coefficient between non-standardized expression profiles. All clusters and related figures were generated using the free open-source statistical software package R. Hierarchical agglomerative clustering using average linkage to measure the dissimilarity between clusters was carried out using the R function hclust from the R cluster library.

The microarray data used here has been validated in our previous study [35] by quantitative real-time PCR (qRT-PCR). For that study, we examined the expression patterns of six genes representing different expression patterns for each of the five consecutive pairs of life stages (egg/infective J2, infective J2/parasitic J2, parasitic J2/J3, J3/J4, J4/female), totaling thirty different genes. The template used for the qRT-PCR was the same biological material used for the microarray hybridizations, and the reactions were performed in technical triplicates. As detailed in [35], we found qualitative agreement for 28 out of 30 tested genes between our GeneChip and qRT-PCR results.

### Identification of signal peptide-encoding *H. glycines *genes

The nucleotide consensus sequences of all 7,530 *H. glycines *probesets (freely available at Affymetrix [54]) were translated into the three forward reading frames (for sense or S1 probesets) or into the three reverse reading frames (for antisense or A1 probesets). Additionally, all nucleotide probeset consensus sequences were translated beginning with the first and second start codons, so that for each probeset up to five translations were generated. All translations were then analyzed for the presence of a signal peptide using SignalP 3.0 [55]. Only those translations were kept for which the *C*-score, *Y*-max, *S*-max, *S*-mean and *D*-score of the SignalP neural network output were positive and for which the SignalP hidden Markov model predicted a signal peptide. We then filtered these translations and kept only those that had at least thirty amino acids after the predicted signal peptide cleavage site. The signal peptides of these translations were cleaved off and the remaining amino acid sequences were checked for the presence of transmembrane helices with the TMHMM software [56]. All translations for which a transmembrane helix was predicted were removed. Probeset nucleotide consensus translations that passed this identification protocol were sorted into probesets, from which a final number of genes was determined by taking probeset variants as designed by Affymetrix into account.

### Identification of cross-hybridizing soybean and *P. sojae *probesets

Soybean and *P. sojae *probesets were considered as cross-hybridizing if their respective signal intensities were called 'present' by Affymetrix' GCOS software (Affymetrix, Santa Clara, CA) in all three replications.

### BLAST searches

All 6,860 unique Affymetrix *H. glycines *gene sequences (contigs) were aligned against the non-redundant GenBank database (downloaded November 2005) using the following parameters in BLASTX with a post-processing cutoff value of 1e-05: filter = seg, lcfilter, W = 4, T = 20, E = 100, B = 25, V = 25, topcomboN = 1, golmax = 10. For Additional data file 1, all *H. glycines *parasitism gene nucleotide sequences (as found in GenBank) were aligned against the non-redundant GenBank database using standard NCBI BLASTX parameters with a post-processing cutoff value of 1e-15 (dated March 2008). To analyze cross-hybridizing *G. max *and *P. sojae *probesets, the respective Affymetrix probeset nucleotide sequences were aligned against the non-redundant GenBank database (downloaded December 2005) using the following parameters in BLASTX with a post-processing cutoff value of 1e-10: filter = seg, lcfilter, W = 4, T = 20, E = 100, B = 10, V = 10, topcomboN = 1, golmax = 10. Additionally, the same *G. max *and *P. sojae *probeset sequences were aligned against the non-redundant GenBank database (downloaded February 2006) using the following parameters in BLASTN with a post-processing cutoff value of 1e-05: M = 1, N = -1, Q = 3, R = 3, lcmask, golmax = 10, topcomboN = 1, filter = seg, B = 100, V = 100, as well as against the 'est_other' database of dbEST (all ESTs other than human and mouse, dated May 12, 2006) using the following parameters in BLASTN with a post-processing cutoff value of 1e-05: M = 1, N = -1, Q = 3, R = 3, lcmask, golmax = 10, topcomboN = 1, filter = seg, and against an in-house cyst nematode nucleotide database (dated March 2006) [35] using the following parameters in BLASTN with a post-processing cutoff value of 1e-05: M = 1, N = -1, Q = 3, R = 3, lcmask, golmax = 10, topcomboN = 1, filter = seg, B = 20, V = 20.

### Identification of plant- or microbe-like *H. glycines *sequences

BLASTX search results (cutoff value 1e-05) of 6,860 Affymetrix contigs against the non-redundant GenBank database were manually sorted into best matches to sequences from plants, phytopathogens/phytosymbionts, soil-living microbes and 'other' microbes. As a counter selection, all matches were removed from further analyses for which a *Caenorhabditis *spp. hit was within 15% of the BLAST score of the best plant or microbe alignment.

### InterProScan analyses

InterProScan was run using InterPro data files dated November 2005 (iprscan_PTHR_DATA_12.0.tar). InterProScan translated all 576 cross-hybridizing soybean and all 134 cross-hybridizing *P. sojae *probesets in six frames and then ran its suite of domain finding tools. We required a minimum translation length of 20 amino acids to be considered by InterProScan, and we used the EGC.0 translation table. Due to the six frames translation, each probeset typically had several alignments amongst the significant open reading frames (ORFs) found in the translation. We kept, as representative of each probeset, the single longest aligning ORF that contained an InterPro domain, even though InterProScan may have found several ORFs for each probeset with alignments to some domain or motif. Results were parsed into files representing expression clusters.

### Sequence alignment and phylogenetic tree for HDA2 sequences

CLUSTAL W [43] was used to align selected HDA2 sequences and to construct a phylogenetic tree. For the phylogenetic tree, the 'neighbor joining' output format from CLUSTAL W was chosen and the 'correct distances' and 'ignore gaps' options were turned off.

### Data

All Affymetrix Soybean Genome Array GeneChip raw and normalized data files analyzed here were deposited previously in the MIAME-compliant ArrayExpress database [57] by these authors [35] and are freely available under accession number E-MEXP-1110.

## Abbreviations

EST: expressed sequence tag; FDR: false discovery rate; HDA2: histone deacetylase-2; HDA19: histone deacetylase-19; infJ2: infective second-stage juvenile; parJ2: parasitic second-stage juvenile; J3: third-stage juvenile; J4: fourth-stage juvenile; ORF: open reading frame; qRT-PCR: quantitative real-time polymerase chain reaction

## Authors' contributions

AAE planned and coordinated the study, analyzed the data and wrote the manuscript. MM and JPM oversaw sequencing and sequence analyses and edited the manuscript. XG and JM assisted in sequence analyses. JR assisted in statistical analyses. ELD provided material. RSH edited the manuscript. DN performed and directed statistical analyses and edited the manuscript. TJB planned the study and edited the manuscript. All authors read and approved the final manuscript.

## Supplementary Material

Additional file 1 **H. glycines parasitome.** Excel file showing *H. glycines *parasitome members, their contigs, probesets, expression cluster membership, mean expression data and q values. Click here for file

Additional file 2 **H. glycines secretome.** Excel file showing *H. glycines *secretome members identified here, their contigs, probesets, expression cluster membership, mean expression data and q values. Click here for file

Additional file 3 **Plant-like *H. glycines *genes.** Excel file showing *H. glycines *plant-like contigs, probesets, expression cluster membership, mean expression data and q values. Click here for file

Additional file 4 **Phytomicrobe-like *H. glycines *genes.** Excel file showing *H. glycines *phytopathogen- and phytosymbiont-like contigs, probesets, expression cluster membership, mean expression data and q values. Click here for file

Additional file 5 **Soilmicrobe-like *H. glycines *genes. **Excel file showing *H. glycines *soilmicrobe-like contigs, probesets, expression cluster membership, mean expression data and q values. Click here for file

Additional file 6 **'Other' microbe-like *H. glycines *genes.** Excel file showing *H. glycines *'other' microbe-like contigs, probesets, expression cluster membership, mean expression data and q values. Click here for file

Additional file 7 **H. glycines parasitism-associated candidate genes.** Excel file showing the combined *H. glycines *genes identified (pooled from additional files 1, 2, 3, 4, 5, 6). Click here for file

Additional file 8 **Expression patterns of plant-like *H. glycines *genes.** TIFF file showing temporal expression patterns of *H. glycines *probesets with highest similarity to plant sequences. All *H. glycines *probesets encoding plant-like proteins were grouped into five expression clusters. The average expression pattern of each cluster is represented by a bold line. Click here for file

Additional file 9 **Expression patterns of phytomicrobe-like *H. glycines *genes. **TIFF file showing temporal expression patterns of *H. glycines *probesets with highest similarity to phytopathogen and phytosymbiont sequences. All *H. glycines *probesets encoding phytopathogen- and phytosymbiont-like proteins were grouped into six expression clusters. The average expression pattern of each cluster is represented by a bold line. Click here for file

Additional file 10 **Expression patterns of soilmicrobe-like *H. glycines *genes.** TIFF file showing temporal expression patterns of *H. glycines *probesets with highest similarity to soilmicrobe sequences. All *H. glycines *probesets encoding soilmicrobe-like proteins were grouped into six expression clusters. The average expression pattern of each cluster is represented by a bold line. Click here for file

Additional file 11 **Expression patterns of 'other' microbe-like *H. glycines *genes.** TIFF file showing temporal expression patterns of *H. glycines *probesets with highest similarity to sequences from 'other' microbe sequences. All *H. glycines *probesets encoding 'other' microbe-like proteins were grouped into six expression clusters. The average expression pattern of each cluster is represented by a bold line. Click here for file

Additional file 12 **HDA2 multiple alignment.** PDF file showing CLUSTAL W multiple alignment for HDA2 homolog sequences, including *H. glycines *(Hg) Affymetrix consensus sequence for probeset HgAffx.19783.1.S1_AT; *C. elegans *(Ce) Wormbase entry CE01472; *Trichinella spiralis *(Ts) ES567375; *Globodera rostochiensis *(Gr) AW506399; *Arabidopsis thaliana *(At) AAM34784.1; *Medicago truncatula *(Mt) EV261025; *Lotus japonicus *(Lj) BW625394. The putative signal peptide-encoding sequence for the *H. glycines *HDA2 is underlined. Click here for file

Additional file 13 **Cross-hybridizing *Glycine max *probesets.** Excel file showing cross-hybridizing *G. max *probesets, expression cluster membership, mean expression data and q values. Click here for file

Additional file 14 **Cross-hybridizing *Phytophthora sojae *probesets.** Excel file showing cross-hybridizing *P. sojae *probesets, expression cluster membership, mean expression data and q values. Click here for file
