# Supplementary figures and images for: Sequence mining and transcript profiling to explore cyst nematode parasitism
Source: BMC Genomics. 2009 Jan 30;10:58. doi: 10.1186/1471-2164-10-58 (PMC2640417; doi:10.1186/1471-2164-10-58)

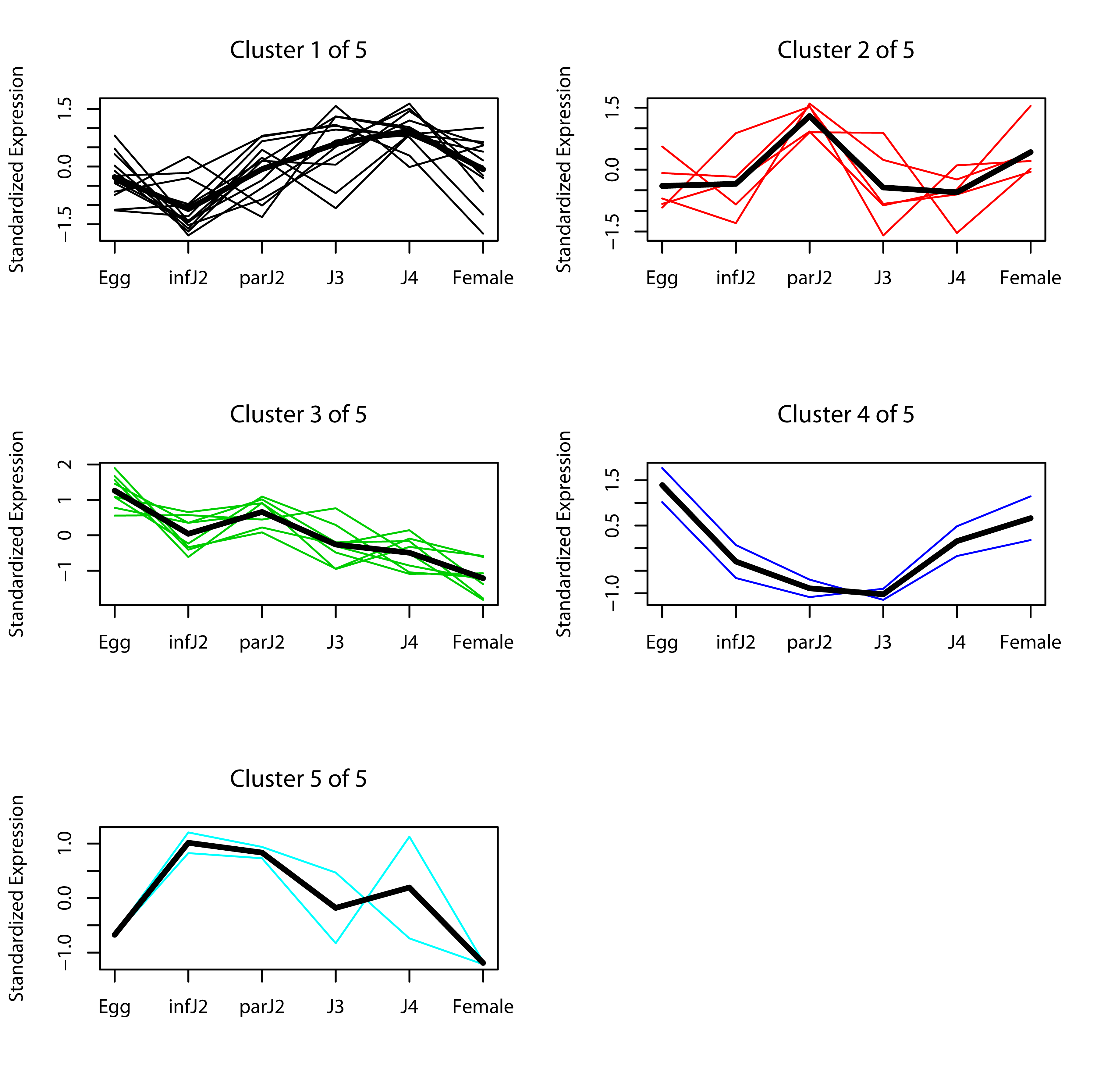

Supplement: Additional file 8 — Expression patterns of plant-like H. glycines genes. TIFF file showing temporal expression patterns of H. glycines probesets with highest similarity to plant sequences. All H. glycines probesets encoding plant-like proteins were grouped into five expression clusters. The average expression pattern of each cluster is represented by a bold line. [file 1471-2164-10-58-S8.tiff]

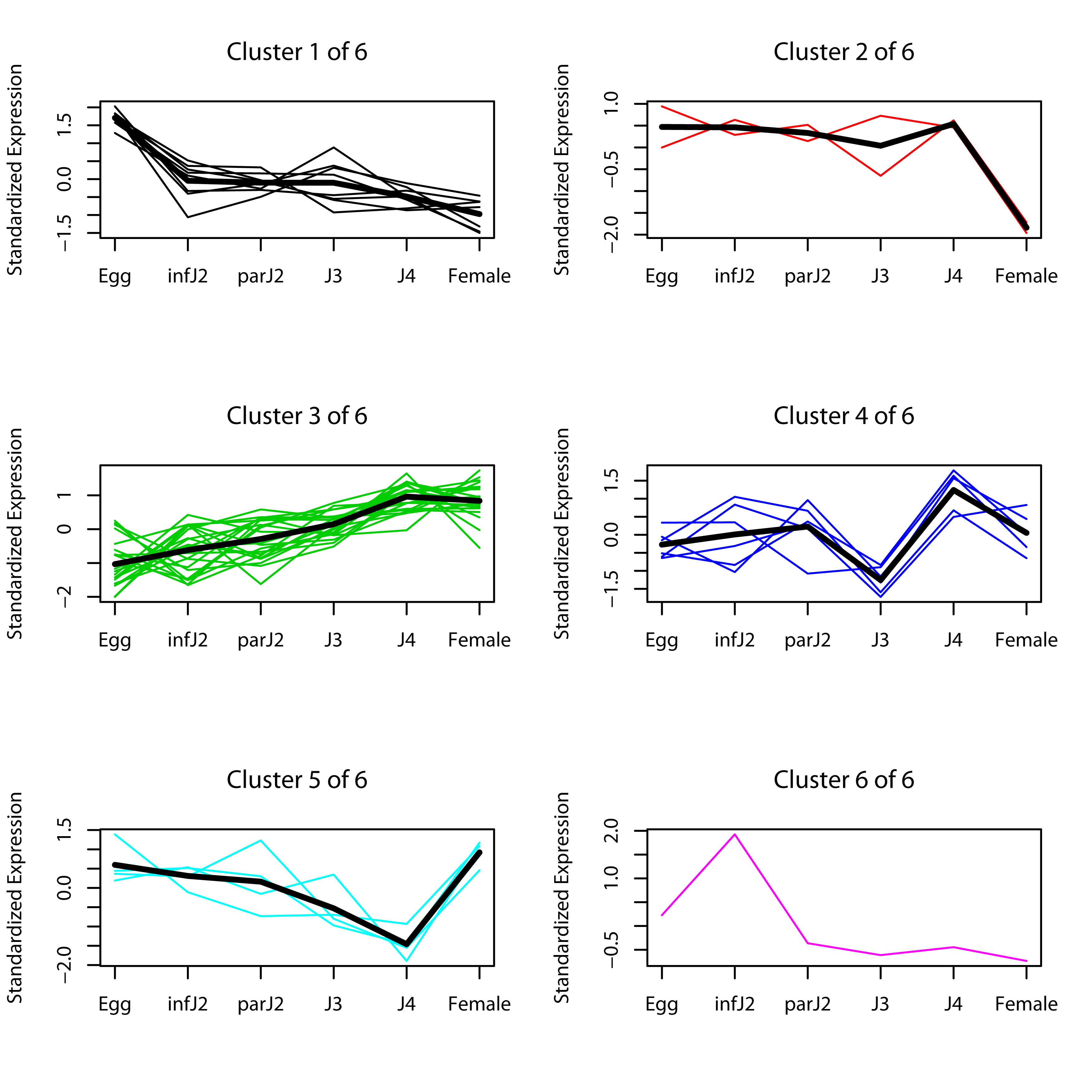

Supplement: Additional file 9 — Expression patterns of phytomicrobe-like H. glycines genes. TIFF file showing temporal expression patterns of H. glycines probesets with highest similarity to phytopathogen and phytosymbiont sequences. All H. glycines probesets encoding phytopathogen- and phytosymbiont-like proteins were grouped into six expression clusters. The average expression pattern of each cluster is represented by a bold line. [file 1471-2164-10-58-S9.tiff]

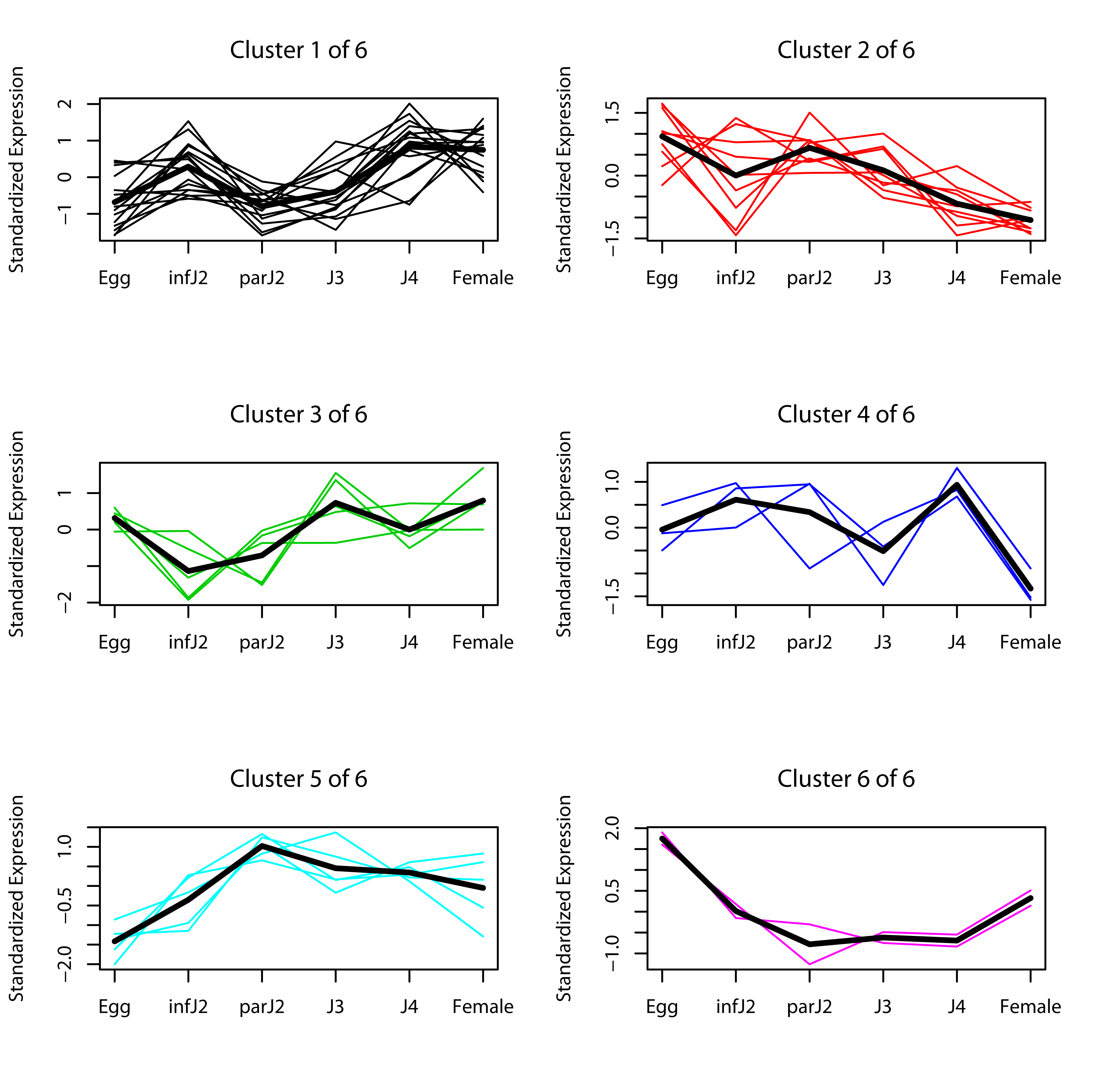

Supplement: Additional file 10 — Expression patterns of soilmicrobe-like H. glycines genes. TIFF file showing temporal expression patterns of H. glycines probesets with highest similarity to soilmicrobe sequences. All H. glycines probesets encoding soilmicrobe-like proteins were grouped into six expression clusters. The average expression pattern of each cluster is represented by a bold line. [file 1471-2164-10-58-S10.tiff]

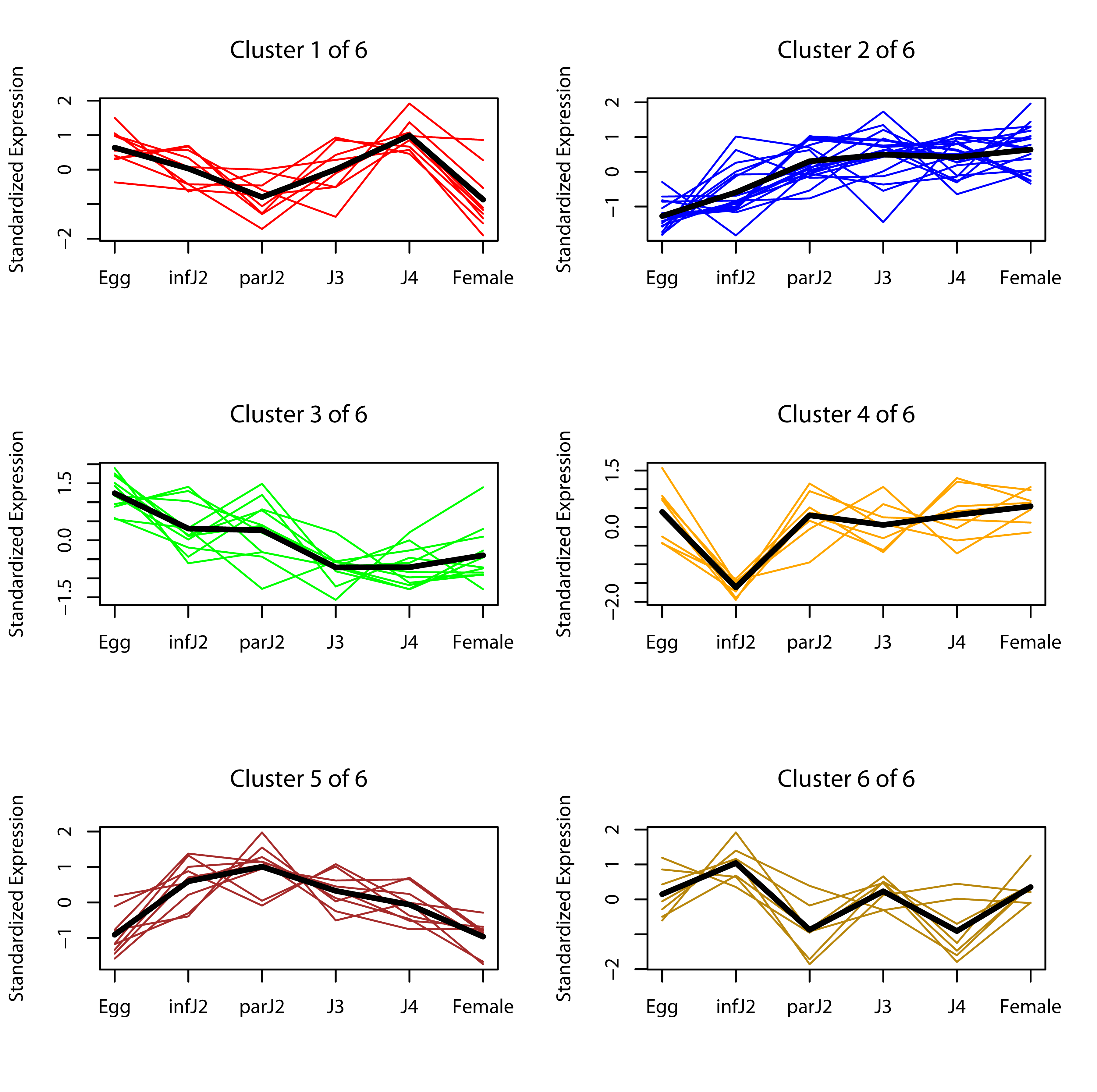

Supplement: Additional file 11 — Expression patterns of 'other' microbe-like H. glycines genes. TIFF file showing temporal expression patterns of H. glycines probesets with highest similarity to sequences from 'other' microbe sequences. All H. glycines probesets encoding 'other' microbe-like proteins were grouped into six expression clusters. The average expression pattern of each cluster is represented by a bold line. [file 1471-2164-10-58-S11.tiff]
