# Supplementary material for: Sequence mining and transcript profiling to explore cyst nematode parasitism
Source: BMC Genomics. 2009 Jan 30;10:58. doi: 10.1186/1471-2164-10-58 (PMC2640417; doi:10.1186/1471-2164-10-58)
Supplement: Additional file 12 — HDA2 multiple alignment. PDF file showing CLUSTAL W multiple alignment for HDA2 homolog sequences, including H. glycines (Hg) Affymetrix consensus sequence for probeset HgAffx.19783.1.S1_AT; C. elegans (Ce) Wormbase entry CE01472; Trichinella spiralis (Ts) ES567375; Globodera rostochiensis (Gr) AW506399; Arabidopsis thaliana (At) AAM34784.1; Medicago truncatula (Mt) EV261025; Lotus japonicus (Lj) BW625394. The putative signal peptide-encoding sequence for the H. glycines HDA2 is underlined. [file 1471-2164-10-58-S12.pdf]

```
Hg     1 -----------------------------LIKAFHLLISLPMCASAALSAFAFLDDSEAE
At     1 ----------------------------------------MATHPEALRRERILNSKLYF
Lj     1 -----------------GPLLLLSPCDSLEPSLPMSSS-SSGTDALALARNRILSSKLYF
Mt     1 IPDNHHRVKSVYRWLCHYFIHPSPPLKYLHRATPMSSPPSCSAADDTLMRNRILSTKLYF
Ce     1 --------------------------------------MSSDKFKLDTLFDDNDEIIEPD
Gr     1 -----------------------------------------------EFLQKHSEMPNVG
Ts     1 ----------------------------------------PRVRGRRSVFKICEVLGIMS

Hg    32 IDWDERCPIVYSEDYNISFFGIEKCHPFDSNKWGNVFRFLIASGALVESAVLRPIEAKKE
At    21 DVPLSKVSIIYSSSYDISFMGIEKLHPFDSSKWGRVCKFLVSDGFLEEKAIVEPLEASKI
Lj    43 DVPPSKVPLIYSESYDISFLGIEKLHPFDSSKWGRICGFLVSFDVLDKKCIVEPLEASKD
Mt    61 DVPPSKVPLIYSDSYDISFLGIEKLHPFDSSKWGRICKFLISFGVLDKNRIVEPLEASKD
Ce    23 GADVKKRNVAYYYHKDVGHFHYGQLHPMKPQRLVVCNDLVVSYEMPKYMTVVESPKLDAA
Gr    14 VQDHYRKRVAYYYDSNVGNYYYGQGHVMKPHRIRMTHHLLLNYGIYRHLDVYRPFPASLE
Ts    21 RTDDSKKRVCYFYDPDIGNYMFSPNHDMKPIRVRMVHCLVYEYQLYRHVTIMEPHRATPD

Hg    92 HLLLVHSKKYLRSLNGRF----TLTRILEVGLVLLFPICLIDRRILRPMRIQTGGTVLAA
At    81 DLLVVHSENYLNSLKSSA----TVARITEVAPVAFFPNFLVQQKVLYPFRKQVGGTILAA
Lj   103 DLLVVHSESYLNSLKESS----NVATIIEVPPVALFPNCLVQNKVLFPFRKQVGGTILAV
Mt   121 DLLVVHSESYLNSLKESS----NVAKIIEVPPVALFPNCLVQRKVLFPFRKQVGGTILAA
Ce    83 DISVFHTEDYVNFLQTVTPKLGLTMPDDVLRQFNIGEDCPIFAGLWDYCTLYAGGSVEGA
Gr    74 DMTRFHSEDYMKFLRNATPEN-LRQFNKQMLKFNVGEDCPVFDGLFEFCQLSSGGSLAAA
Ts    81 EMKIFHDEDYIDMLRRAKPEH-MNCNLDALSYYNIGDDCPLFDGIFEYCQITTGGSIAGA

Hg   148 RVSLIR--GWAINIGGGFHHASEGKGGGFCVYADVTLAIKLLFANELIKSAMIVDVDAHQ
At   137 KLATER--GWAINIGGGFHHCTAERGGGFCAFADISLCIHFAFLRLRISRVMIIDLDAHQ
Lj   159 KLAKER--GWAINVGGGFHHCSAE------------------------------------
Mt   177 KLAKER--GWAINVGGGFHHCSAENGGGFCAYADISLCVHFAFVQLNISRVIIIDLDAH-
Ce   143 RRLNHKMNDIVINWPGGLHHAKKSEASGFCYVNDIVLGILELLK--YHKRVLYIDIDIHH
Gr   133 VKLNKQKAEIAINWMGGLHHAKKSEASGFCYTNDIVLGILELLK--YHKRVLYVDIDVHH
Ts   140 QSLNSGEYDIAINWAGGMHHAKWYKASGFCFVNDIVLAILQLLK--KHQRVLYVDIDCHM

Hg   206 GNGHETDFASDSRVYILDL-----------------------------------------
At   195 GNGHETDLGDDNRVYILDMYNPEIYPFDYRAARRFIDQKVEVMSGTTTDEYLRKLDEALEV
Lj       ------------------------------------------------------------
Mt       ------------------------------------------------------------
Ce   201 GDGVQEAFNNSDRVMTVSFHRFG-QYFPGSGSIMDKGVGPGKYFAINVPLMAAIRDEPYL
Gr   191 GDGVEEAFYTTDRVMTVSVSQIPAIFFPGTGELKVIKFL--MNSFLG-------------
Ts   198 GMGVEEAFYTTDRVMTISFHKFG-NFFPGTGNYDSIGYGKGLHYAVNFPLH---------

Hg       ------------------------------------------------------------
At   255 ASRN---------FQPELVIYNAGTDILDGDPLGLLKISPDGITSRDEKVFRFAREKNIP
Lj       ------------------------------------------------------------
Mt       ------------------------------------------------------------
Ce   260 KLFESVISGVEENFNPEAIVLQCGSDSLCEDRLGQFALSFNAHARAVKYVKSLGKPLMVL
Gr       ------------------------------------------------------------
Ts       ------------------------------------------------------------

Hg       ------------------------------------------------------------
At   306 LVMLTSGGYMKSSARVIADSIENLSRQGLIQTRPE-------------------------
Lj       ------------------------------------------------------------
Mt       ------------------------------------------------------------
Ce   320 GGGGYTLRNVARCWALETGVILGLRMDDEIPGTSLYSHYFTPRLLRPNLVPKMNDANSAA
Gr       ------------------------------------------------------------
Ts       ------------------------------------------------------------

Hg       ------------------------------------------------------------
At       ------------------------------------------------------------
Lj       ------------------------------------------------------------
Mt       ------------------------------------------------------------
Ce   380 YLASIEKETLACLRMIRGAPSVQMQNIVGIRLDEIEQIEENERLQKSSKSSIEYEVGKVS
Gr       ------------------------------------------------------------
Ts       ------------------------------------------------------------
```
